# Supplementary material for: Filovirus infection disrupts epithelial barrier function and ion transport in human iPSC-derived gut organoids
Source: PLoS Pathog. 2025 Nov 24;21(11):e1013698. doi: 10.1371/journal.ppat.1013698 (PMC12698023; doi:10.1371/journal.ppat.1013698)
Supplement: S1 Table — Detailed list of all components used in the culture medium for establishing and maintaining primary colonic organoids. The table includes reagent name, final concentration, supplier, catalog number, and notes on growth factor or supplement function where applicable. (DOCX) [file ppat.1013698.s001.docx]

S1 Table. Composition of culture medium for primary colonic organoids (PCOs). Detailed list of all components used in the culture medium for establishing and maintaining primary colonic organoids.

| **Splitting Medium**: 500 mL DMEM/F12, 10% FBS, 1% Penicillin/Streptomycin |
| --- |
| **DF20**: 500 mL DMEM/F12, 20% FBS, 1% Penicillin/Streptomycin |
| **R-spondin selection medium:** 500 mL Advanced DMEM + 50 mL FBS + 5 mL Penicillin/Streptomycin + 5 mL Glutamax + 1.5 mL Zeocin |
| **R-spondin collection medium:** 500 mL DMEM/F12 + 5 mL Glutamax. Note no Penicillin/Streptomycin |
| **R-spondin freezing medium:** 5 mL selection medium + 3 mL FBS + 2 mL DMSO |
| **WNT selection medium:** 500 mL Advanced DMEM + 50 mL FBS + 2 mL G418 (100mg/mL). Note no Penicillin/Streptomycin. |
| **WNT collection medium:** 500 mL Advanced DMEM + 50 mL FBS. Note no Penicillin/Streptomycin |
| **WNT Freezing medium:** 5 mL WNT selection medium + 3 mL FBS + 2 mL DMSO |
