# Supplementary material for: Filovirus infection disrupts epithelial barrier function and ion transport in human iPSC-derived gut organoids
Source: PLoS Pathog. 2025 Nov 24;21(11):e1013698. doi: 10.1371/journal.ppat.1013698 (PMC12698023; doi:10.1371/journal.ppat.1013698)
Supplement: S2 Table — Comprehensive list of all antibodies, dyes, and reagents used in this study. Information includes target antigen, host species, clone or catalog number, source or supplier, application (e.g., IF, IHC, FACS), and dilution. (DOCX) [file ppat.1013698.s002.docx]

S2 Table. Antibodies, dyes, and reagents used for immunofluorescence, immunohistochemistry, and flow cytometry. Comprehensive list of all antibodies, dyes, and reagents used in this study. Information includes target antigen, host species, clone or catalog number, source or supplier, application (e.g., IF, IHC, FACS), and dilution.

| **Antibodies/Dyes, and Reagents for Immunostaining and FACS** | | |
| --- | --- | --- |
| **Target** | **Vendor** | **Catalog No** |
| **FACS** | | |
| Anti-human CD26 PE Conjugate | BioLegend | 302705 |
| Calcein Blue | Life Technologies | C1429 |
| Anti-human CXCR4 - PE | ThermoFisher | MHCXCR404 |
| Anti-human c-Kit - APC | BioLegend | 323205 |
| Zombie NIR Fixable Viability Kit | BioLegend | 423105 |
| Rabbit anti-EBOV NP | IBT | 0301-012 |
| **IFA** | | |
| 4% normal goat serum (NGS) | Jackson ImmunoResearch | 005-000-121 |
| 13 mm cover slips | ThermoFisher Scientific | 174950 |
| ProLong Diamond Antifade Mountant, | ThermoFisher Scientific | P36961 |
| BRAND cavity slides | Millipore Sigma | BR475505 |
| **Primary antibodies** | | |
| Chicken anti- GFP IgY (1:200) | ThermoFisher Scientific | A10262 |
| Mouse anti-Villin (1:200) | Millipore | MAB1671 |
| Mouse anti-MUC2 (1:200) | SantaCruz | sc-515032 |
| Rabbit anti-EBOV NP (1:200) | IBT | 0301-012 |
| Rabbit anti-MARV NP (1:500) | IBT | 0303-012 |
| Rabbit anti-MARV NC (1:200) | Mühlberger lab |  |
| **Secondary antibodies** | | |
| Donkey anti-chicken AF488 (1:500) | Jackson Immunoresearch Labs | 703-545-155 |
| Donkey anti-rabbit Alexa Fluor 594 IgG (H+L) (1:500) | ThermoFisher Scientific | A-21207 |
| Donkey anti-mouse Alexa Fluor 488 IgG (H+L) (1:500) | ThermoFisher Scientific | A-21202 |
| Hoechst 33342 (1:2000) | ThermoFisher Scientific | 62249 |
