# Supplementary material for: Filovirus infection disrupts epithelial barrier function and ion transport in human iPSC-derived gut organoids
Source: PLoS Pathog. 2025 Nov 24;21(11):e1013698. doi: 10.1371/journal.ppat.1013698 (PMC12698023; doi:10.1371/journal.ppat.1013698)
Supplement: S3 Table — List of primary and secondary antibodies used for IHC staining of primary and induced pluripotent stem cell–derived gut organoids. The table includes target antigen, antibody host species, clone or catalog number, supplier, dilution, and application details. (DOCX) [file ppat.1013698.s003.docx]

S3 Table. Antibodies used for immunohistochemistry (IHC) analysis. List of primary and secondary antibodies used for IHC staining of primary and induced pluripotent stem cell–derived gut organoids. The table includes target antigen, antibody host species, clone or catalog number, supplier, dilution, and application details.

Chromogranin A + EBOV VP35 IHC

| Sequence | Antigen Target | Species origin | Clone | Manufacturer | Catalog | Dilution | Incubation | Fluorophore | Fluorophore dilution |
| --- | --- | --- | --- | --- | --- | --- | --- | --- | --- |
| 1 | Chromo-granin A | Rb | Polyclonal | Abcam | Ab15160 | 1:500 | 37°C, 40 min | Opal 570 | 1:100 |
| 2 | EBOV VP35 | Ms | 10C7 | Kera-fast | EMS703 | 1:2500 | 37°C, 32 min | Opal 690 | 1:200 |

Lysozyme + EBOV VP35 IHC

| Sequence | Antigen Target | Species origin | Clone | Manufacturer | Catalog | Dilution | Incubation | Fluorophore | Fluorophore dilution |
| --- | --- | --- | --- | --- | --- | --- | --- | --- | --- |
| 1 | Lysozyme | Rb | Polyclonal | Invitrogen | PA1-289680 | 1:100 | 37°C, 40 min | Opal 570 | 1:100 |
| 2 | EBOV VP35 | Ms | 10C7 | Kera-fast | EMS703 | 1:2500 | 37°C, 32 min | Opal 690 | 1:200 |

Muc2 + EBOV VP35 IHC

| Sequence | Antigen Target | Species origin | Clone | Manufacturer | Catalog | Dilution | Incubation | Fluorophore | Fluorophore dilution |
| --- | --- | --- | --- | --- | --- | --- | --- | --- | --- |
| 1 | Villin | CST | ID2C3 | CST | 55883 | 1:100 | 37°C, 40 min | Opal 570 | 1:100 |
| 2 | EBOV VP35 | Ms | 10C7 | Kerafast | EMS703 | 1:2500 | 37°C, 32 min | Opal 690 | 1:200 |

Villin + EBOV VP35 IHC

| Sequence | Antigen Target | Species origin | Clone | Manufacturer | Catalog | Dilution | Incubation | Fluorophore | Fluorophore dilution |
| --- | --- | --- | --- | --- | --- | --- | --- | --- | --- |
| 1 | Villin | CST | ID2C3 | CST | 55883 | 1:100 | 37°C, 40 min | Opal 570 | 1:100 |
| 2 | EBOV VP35 | Ms | 10C7 | Kerafast | EMS703 | 1:2500 | 37°C, 32 min | Opal 690 | 1:200 |

2CXCL10 ISH + EBOV VP35 IHC

| Sequence | Antigen Target | Species origin | Clone | Manufacturer | Catalog | Dilution | Incubation | Fluorophore | Fluorophore dilution |
| --- | --- | --- | --- | --- | --- | --- | --- | --- | --- |
| 1 | Hs-CXCL10 | N/A | N/A | ACD | 311859 | Predilute | 43°C, 2 hrs | Opal 570 | 1:225 |
| 2 | EBOV VP35 | Ms | 10C7 | Kera-fast | EMS703 | 1:2500 | 37°C, 32 min | Opal 690 | 1:200 |

IFNB ISH + EBOV VP35 IHC

| Sequence | Antigen Target | Species origin | Clone | Manufacturer | Catalog | Dilution | Incubation | Fluorophore | Fluorophore dilution |
| --- | --- | --- | --- | --- | --- | --- | --- | --- | --- |
| 1 | Hs-IFNB1 | N/A | N/A | ACD | 417079 | Predilute | 43°C, 2 hrs | Opal 570 | 1:150 |
| 2 | EBOV VP35 | Ms | 10C7 | Kerafast | EMS703 | 1:2500 | 37°C, 32 min | Opal 690 | 1:200 |

MX1 ISH + EBOV VP35 IHC

| Sequence | Antigen Target | Species origin | Clone | Manufacturer | Catalog | Dilution | Incubation | Fluorophore | Fluorophore dilution |
| --- | --- | --- | --- | --- | --- | --- | --- | --- | --- |
| 1 | Hs-MX1 | N/A | N/A | ACD | 403839 | Predilute | 43°C, 2 hrs | Opal 570 | 1:100 |
| 2 | EBOV VP35 | Ms | 10C7 | Kerafast | EMS703 | 1:2500 | 37°C, 32 min | Opal 690 | 1:200 |

CXCL10 ISH + MARV GP

| Sequence | Antigen Target | Species origin | Clone | Manufacturer | Catalog | Dilution | Incubation | Fluorophore | Fluorophore dilution |
| --- | --- | --- | --- | --- | --- | --- | --- | --- | --- |
| 1 | Hs-CXCL10 | N/A | N/A | ACD | 311859 | Predilute | 43°C, 2 hrs | Opal 570 | 1:225 |
| 2 | MARV GP | Rb | Polyclonal | IBT | 0303-007 | 1:1500 | RT, 2 hrs | Opal 690 | 1:200 |

IFNB ISH + MARV GP

| Sequence | Antigen Target | Species origin | Clone | Manufacturer | Catalog | Dilution | Incubation | Fluorophore | Fluorophore dilution |
| --- | --- | --- | --- | --- | --- | --- | --- | --- | --- |
| 1 | Hs-IFNB1 | N/A | N/A | ACD | 417079 | Predilute | 43°C, 2 hrs | Opal 570 | 1:150 |
| 2 | MARV GP | Rb | Polyclonal | IBT | 0303-007 | 1:1500 | RT, 2 hrs | Opal 690 | 1:200 |

MX1 ISH + MARV GP

| Sequence | Antigen Target | Species origin | Clone | Manufacturer | Catalog | Dilution | Incubation | Fluorophore | Fluorophore dilution |
| --- | --- | --- | --- | --- | --- | --- | --- | --- | --- |
| 1 | Hs-MX1 | N/A | N/A | ACD | 403839 | Predilute | 43°C, 2 hrs | Opal 570 | 1:100 |
| 2 | MARV GP | Rb | Polyclonal | IBT | 0303-007 | 1:1500 | RT, 2 hrs | Opal 690 | 1:200 |

CXCL10 ISH + EBOV VP35/MARV GP (run on naïve organoids)

| Sequence | Antigen Target | Species origin | Clone | Manufacturer | Catalog | Dilution | Incubation | Fluorophore | Fluorophore dilution |
| --- | --- | --- | --- | --- | --- | --- | --- | --- | --- |
| 1 | Hs-CXCL10 | N/A | N/A | ACD | 311859 | Predilute | 43°C, 2 hrs | Opal 570 | 1:225 |
| 2 | EBOV VP35 | Ms | 10C7 | Kerafast | EMS703 | 1:2500 | 37°C, 32 min | Opal 690 | 1:200 |
| 3 | MARV GP | Rb | Polyclonal | IBT | 0303-007 | 1:1500 | RT, 2 hrs | Opal 780 | 1:40 |

IFNB + EBOV VP35/MARV GP (run on naïve organoids)

| Sequence | Antigen Target | Species origin | Clone | Manufacturer | Catalog | Dilution | Incubation | Fluorophore | Fluorophore dilution |
| --- | --- | --- | --- | --- | --- | --- | --- | --- | --- |
| 1 | Hs-IFNB1 | N/A | N/A | ACD | 417079 | Predilute | 43°C, 2 hrs | Opal 570 | 1:150 |
| 2 | EBOV VP35 | Ms | 10C7 | Kerafast | EMS703 | 1:2500 | 37°C, 32 min | Opal 690 | 1:200 |
| 3 | MARV GP | Rb | Polyclonal | IBT | 0303-007 | 1:1500 | RT, 2 hrs | Opal 780 | 1:40 |

MX1 + EBOV VP35/MARV GP (run on naïve organoids)

| Sequence | Antigen Target | Species origin | Clone | Manufacturer | Catalog | Dilution | Incubation | Fluorophore | Fluorophore dilution |
| --- | --- | --- | --- | --- | --- | --- | --- | --- | --- |
| 1 | Hs-MX1 | N/A | N/A | ACD | 403839 | Predilute | 43°C, 2 hrs | Opal 570 | 1:100 |
| 2 | EBOV VP35 | Ms | 10C7 | Kerafast | EMS703 | 1:2500 | 37°C, 32 min | Opal 690 | 1:200 |
| 3 | MARV GP | Rb | Polyclonal | IBT | 0303-007 | 1:1500 | RT, 2 hrs | Opal 780 | 1:40 |
