# Supplementary figures and images for: Filovirus infection disrupts epithelial barrier function and ion transport in human iPSC-derived gut organoids
Source: PLoS Pathog. 2025 Nov 24;21(11):e1013698. doi: 10.1371/journal.ppat.1013698 (PMC12698023; doi:10.1371/journal.ppat.1013698)

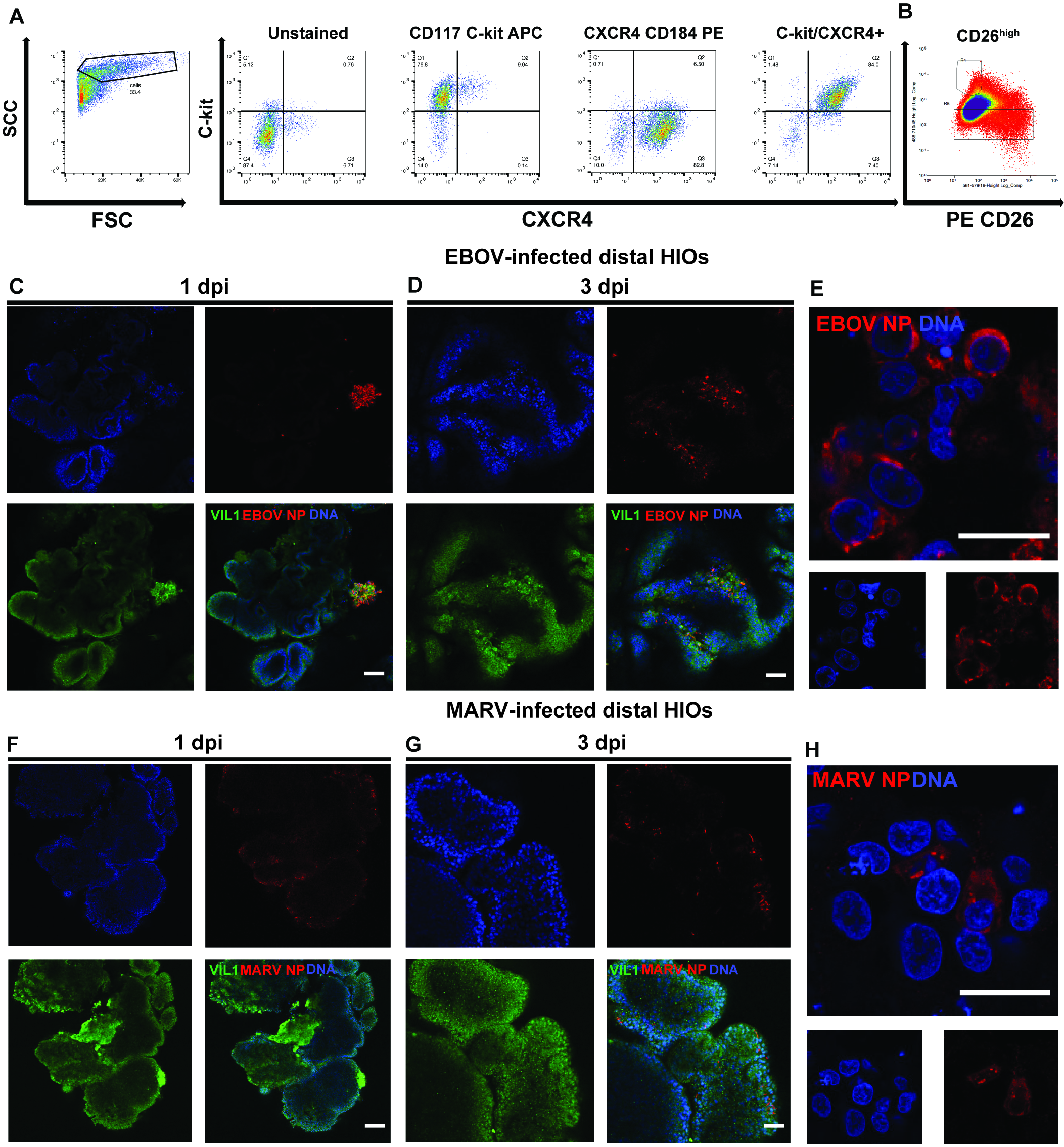

Supplement: S1 Fig — (A) BU310-Cre2 iPSCs were differentiated into definitive endoderm by day 3, followed by specification into CD26 ⁺ gut progenitors by day 15. (B) Gut progenitors were isolated by fluorescence-activated cell sorting (FACS) and cultured in CKDCI medium to generate HCOs. On day 35 of differentiation, HCOs were infected with EBOV or MARV at a MOI of 10. (C–H) Confocal microscopy was performed at 1 and 3 dpi to assess viral replication. Immunofluorescence staining was conducted using antibodies against viral nucleoproteins (NP for EBOV and NC for MARV; red), villin1 to mark intestinal epithelial cells (green), and Hoechst for nuclear counterstaining (blue). Images were acquired using a Zeiss LSM 710 Live-Duo confocal microscope with two-photon capability. Panels C, D, F, and G: scale bars = 100 μm; panels E and H: scale bars = 10 μm. Data shown are representative of three independent infection experiments (n = 3). (TIF) [file ppat.1013698.s004.tif]

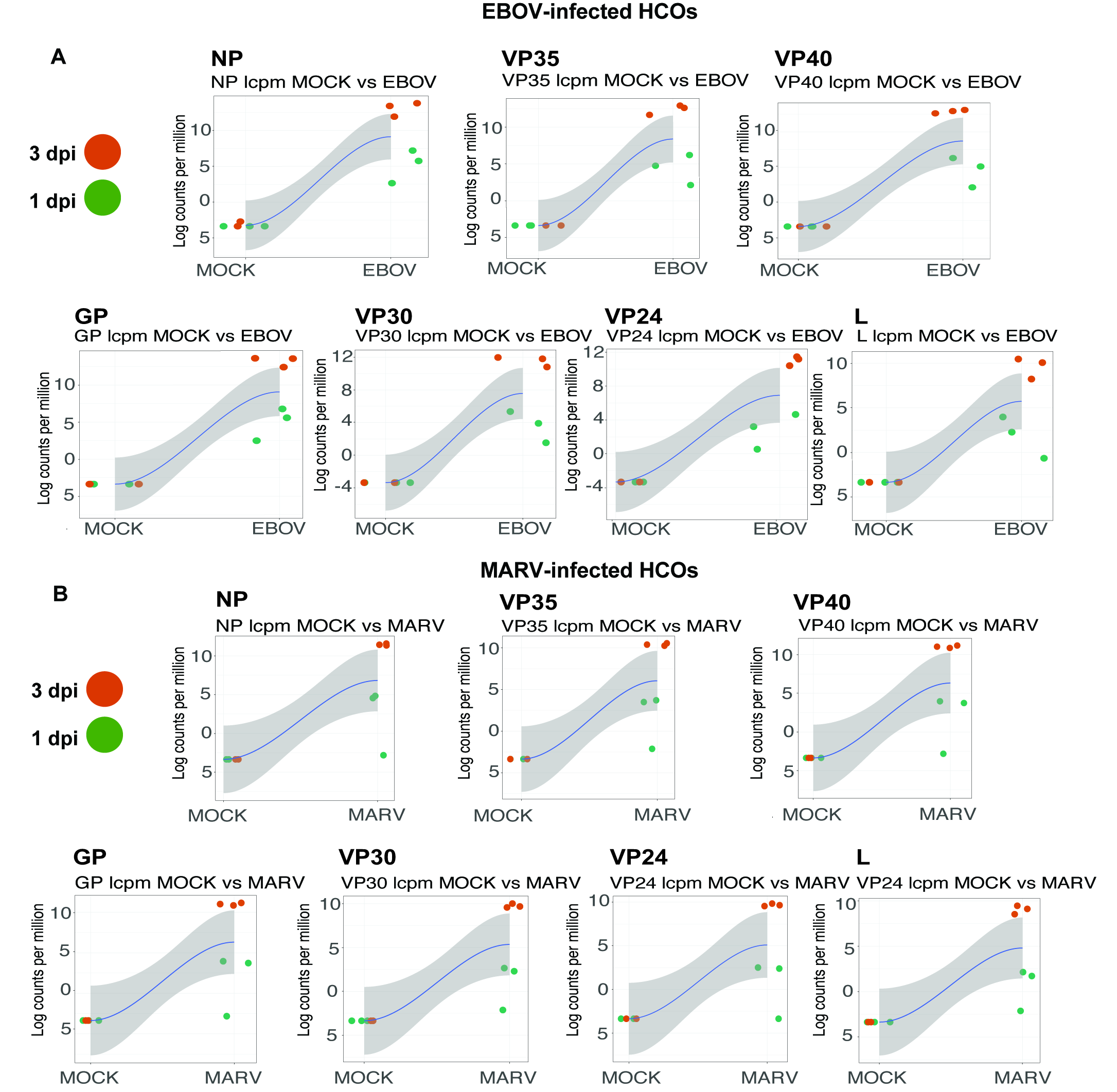

Supplement: S2 Fig — Loess-smoothed plots showing the relative abundance of viral transcripts in human colonic organoids (HCOs) infected with EBOV or MARV compared to mock-infected controls at 1 and 3dpi. (A) EBOV-infected versus mock-infected HCOs. (B) MARV-infected versus mock-infected HCOs. Data are representative of n = 3 independent infections per condition. (TIF) [file ppat.1013698.s005.tif]

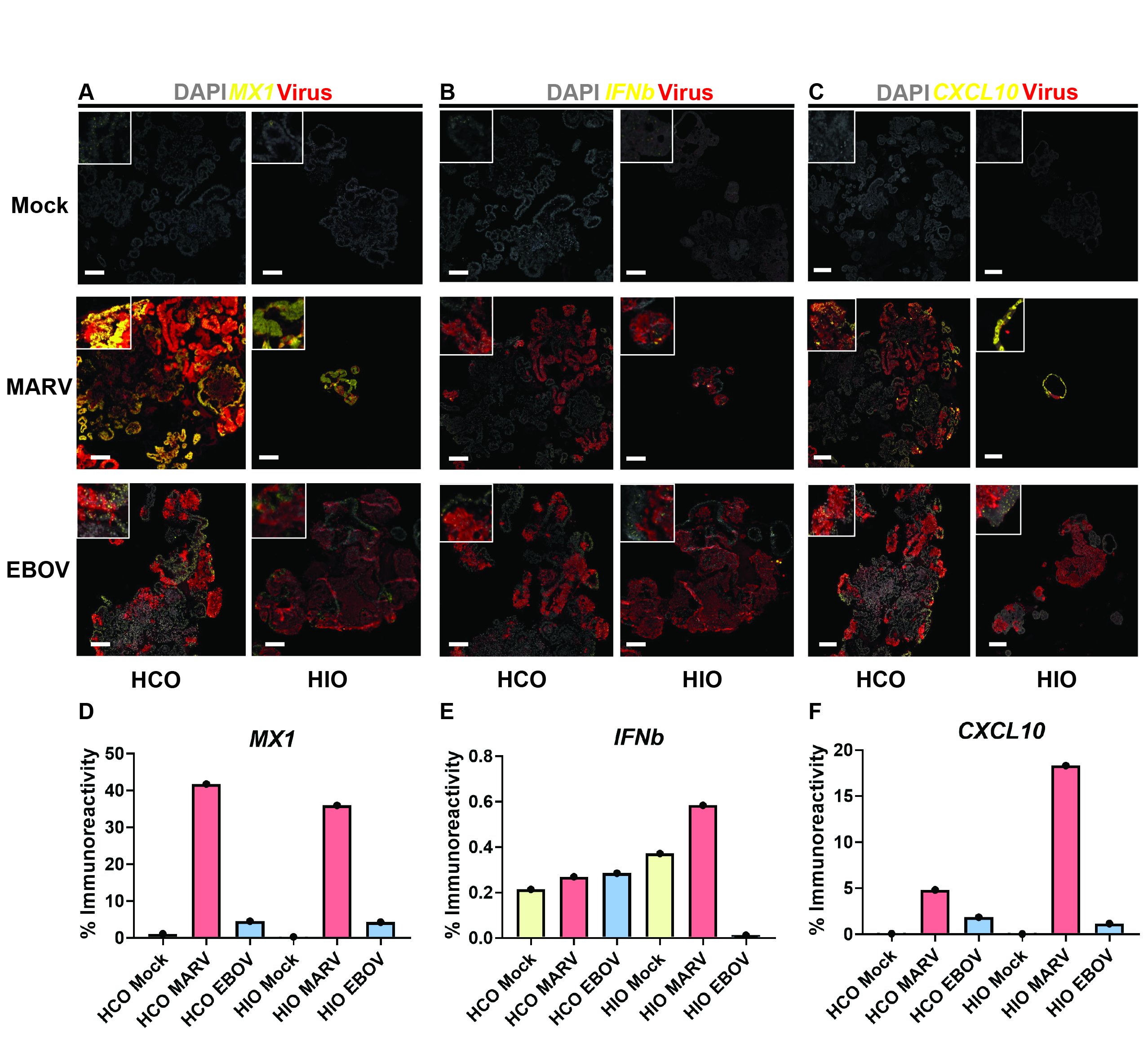

Supplement: S3 Fig — (A–C) Representative combined IHC and ISH images of distal HCOs and proximal HIOs infected with MARV or EBOV at an MOI of 10 and harvested at 3 dpi. Staining was performed using ISH probes targeting the mRNA of human interferon-stimulated genes (ISGs) MX1 (A), IFNb (B), and CXCL10 (C) (yellow), and antibodies targeting EBOV VP35 or GP MARV, respectively (red), with DAPI for nuclear counterstaining (gray). (D–F) Quantification of immune-reactive positive pixel area for MX1 (D), IFNb (E), and CXCL10 (F) mRNA expression. Data are representative of two independent infections (n = 3). Scale bars = 200 μm. (TIF) [file ppat.1013698.s006.tif]

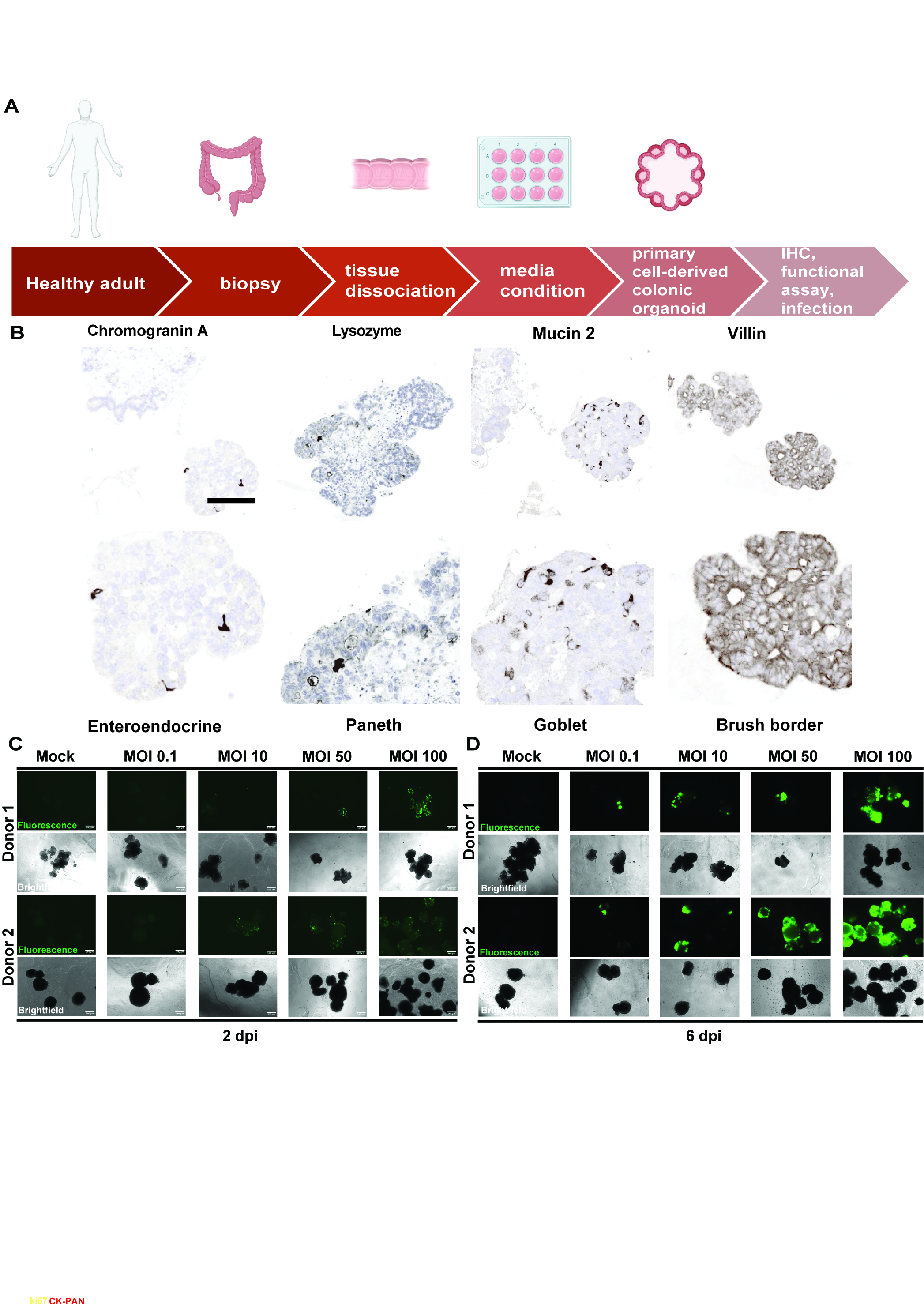

Supplement: S4 Fig — (A) Schematic illustrating the experimental protocol for primary cell-derived human intestinal organoids (PCOs). Created in BioRender. Muhlberger, E. (2025) https://BioRender.com/ev6enkh. (B) Immunohistochemical analysis confirms the presence of lysozyme-positive Paneth cells, chromogranin A-positive enteroendocrine cells, mucin 2-positive goblet cells, and expression of the apical brush border protein villin in PCOs. Hematoxylin staining (blue) and antibody-based staining (brown) are shown. Scale bar = 100 µm. (C and D) Organoids were infected with EBOV-ZsGreen at varying MOIs, 0.1, 10, 50 and 100 and imaged at 2 and 6 dpi. Images were captured using the EVOS M50000 Imaging System. Exposure time for fluorescence images was 25 ms. Scale bar = 100 µm. (TIF) [file ppat.1013698.s007.tif]

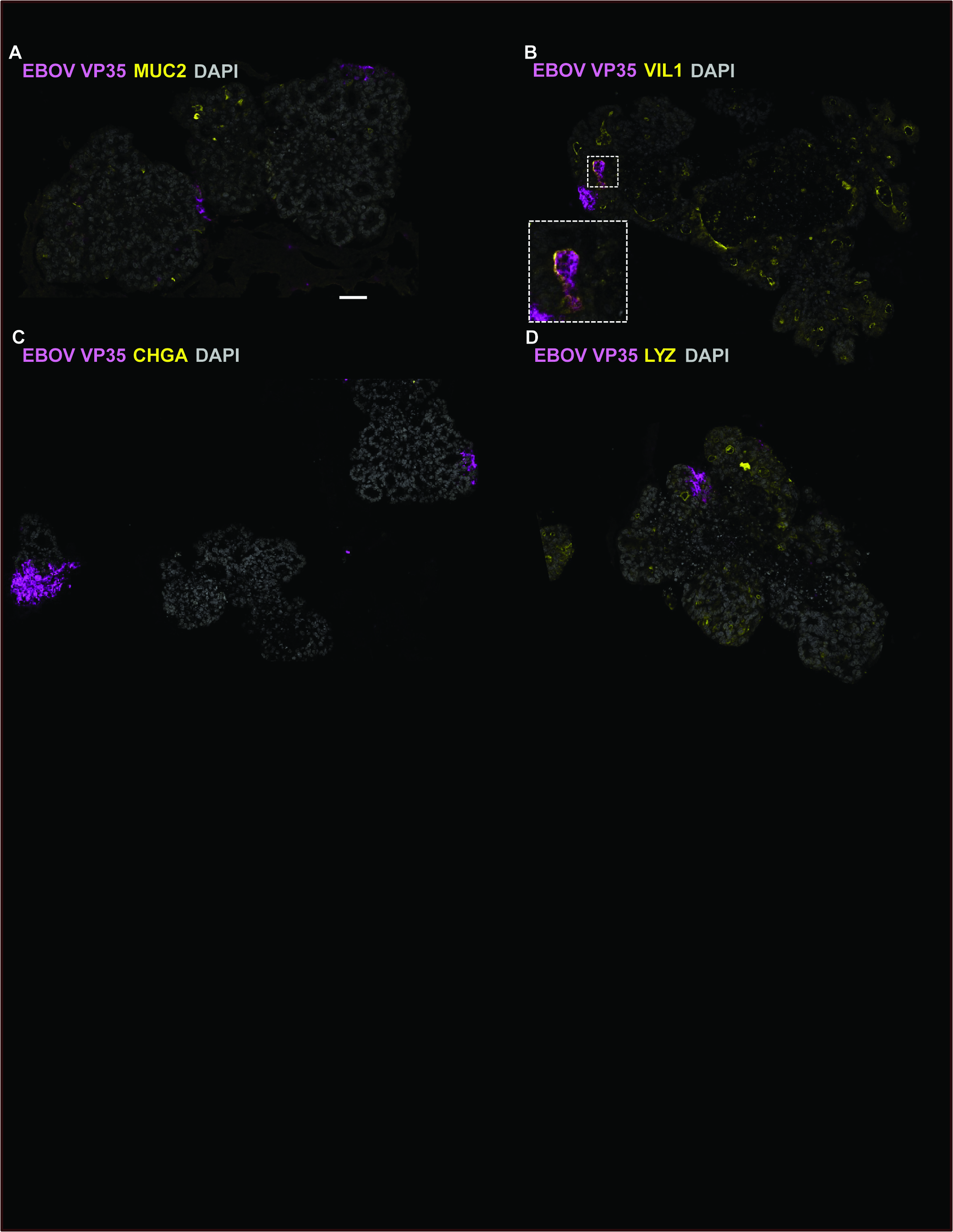

Supplement: S5 Fig — PCOs were infected with EBOV at an MOI of 10. At 3 dpi, cells were fixed with 4% PFA and processed for staining using antibodies against EBOV VP35 and cell-type-specific intestinal epithelial markers. Colocalization of EBOV VP35 (yellow) with intestinal epithelial cell markers in 2D sections of primary cell-derived intestinal organoids. Intestinal epithelial cell types were identified using specific markers: (A) MUC2 (magenta, goblet cells), (B) VIL (magenta, brush border protein), (C) CHGA (magenta, enteroendocrine cells), and (D) LYZ (magenta, Paneth cells), with nuclei stained using DAPI (grey). Inset in (B) shows a magnified view of the apical brush border to highlight colocalization. Scale bar = 100 µm. (TIF) [file ppat.1013698.s008.tif]

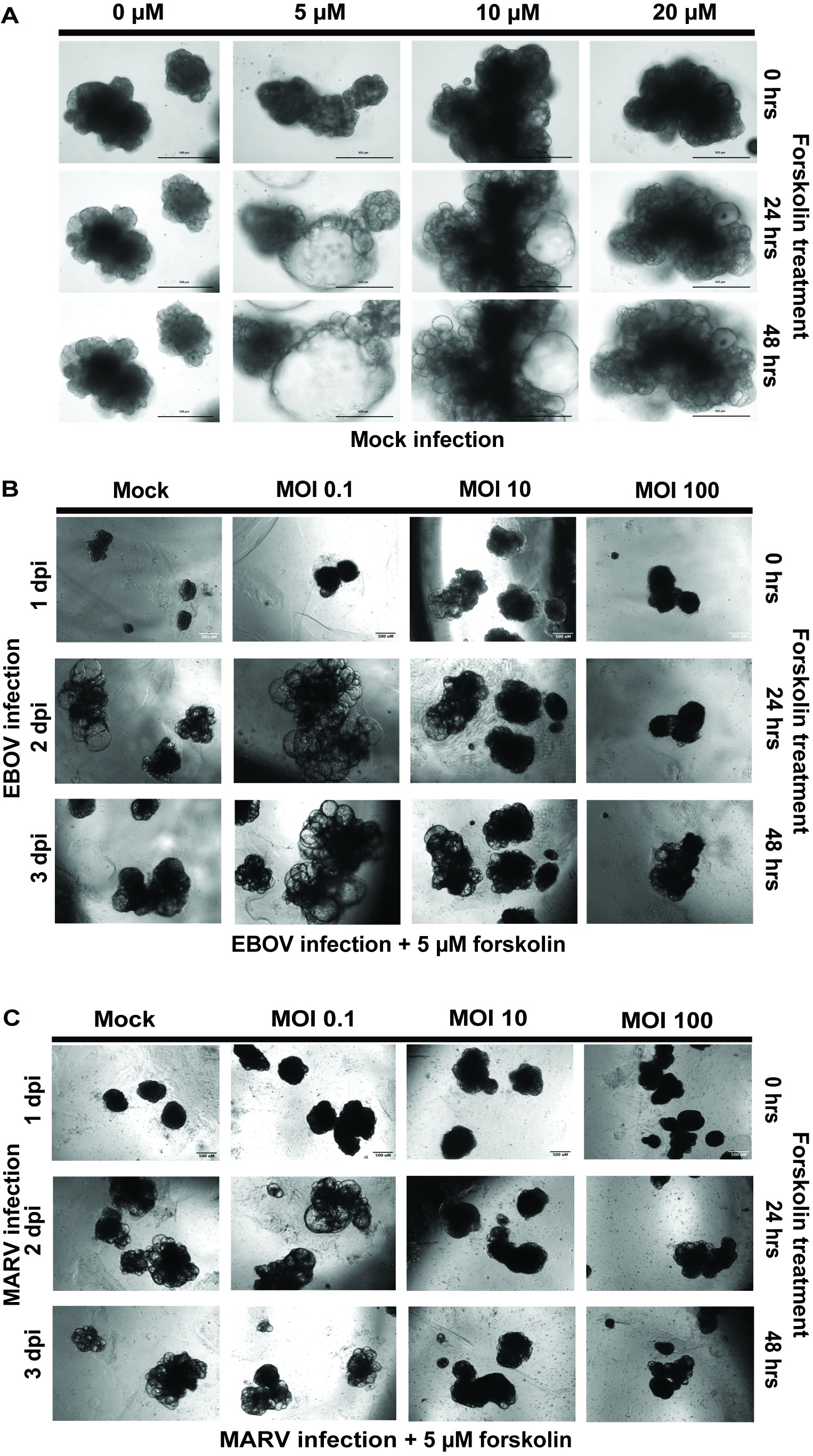

Supplement: S6 Fig — (A) At day 35 of differentiation, organoids were treated with forskolin at concentrations of 5, 10, and 20 µM (dissolved in DMSO) and imaged at 0 hours (top), 24 hours (middle), and 48 hours (bottom) to assess forskolin-induced swelling. Images were acquired using a Keyence BZ-X710 fluorescence microscope. Scale bar = 500 µm. (B and C) PCOs were infected with (B) EBOV or (C) MARV at MOI 0.1, 10, or 100. At 1 dpi, organoids were treated with 5 µM forskolin and subsequently imaged at 0 hours (1 dpi), 24 hours (2 dpi), and 48 hours (3 dpi) post-treatment. Changes in organoid morphology, including swelling and structural integrity, were evaluated at each time point. Images were captured using the EVOS M5000 Imaging System. Scale bar = 500 µm. (TIF) [file ppat.1013698.s009.tif]
