# Supplementary material for: Filovirus infection disrupts epithelial barrier function and ion transport in human iPSC-derived gut organoids
Source: PLoS Pathog. 2025 Nov 24;21(11):e1013698. doi: 10.1371/journal.ppat.1013698 (PMC12698023; doi:10.1371/journal.ppat.1013698)
Supplement: Attachment — Submitted filename: Flores Response to reviewers without editors.docx [file ppat.1013698.s011.docx]

**Response to Reviewers' Comments**

**Part II – Major Issues: Key Experiments Required for Acceptance**
Reviewer #1: The authors have developed gastrointestinal organoid models for filovirus infection and employed multiple assays to characterize host responses. While they present transcriptomic data alongside in situ hybridization data, it remains unclear whether these transcriptional changes translate into secretion of cytokines, chemokines, or interferons. Can ELISA or similar assays detect these secreted proteins in the gastrointestinal organoid system?

**Response:** This is an excellent suggestion, and we regret that we did not think about it when we performed the experiments. Unfortunately, due to BSL-4 biosafety constraints and the design of the original experiments, supernatants were not collected or stored, and thus retrospective analysis is not feasible. For this reason, we cannot perform additional cytokine or multiplex analysis. We acknowledge that such data would provide additional insights, but repeating the experiments to collect the supernatants would be cost- and time-prohibitive. That said, we will consider incorporating this approach in future studies to enhance the depth of our analysis and are grateful for this valuable comment.

Reviewer #2: (No Response)

Reviewer #3: 1. The authors appear to use only one donor for their iPSC-derived organoids and only two for their primary cell-derived organoids. Given donor-to-donor variability, which is a known issue with organoids and primary cultures, biases cannot be excluded. Therefore, it is absolutely needed to verify all results in at least 3 different donors.

**Response:** We appreciate the reviewer’s emphasis on the importance of donor-to-donor variability, which is a critical and well-documented consideration in the organoid and iPSC fields. We respectfully clarify that our study in fact used two genetically distinct iPSC donors, as well as two distinct primary tissue donors for validation. Each iPSC line underwent three or more independent differentiations per experiment, ensuring biological replication and enabling assessment of reproducibility within lines.

We fully recognize that the use of more independent donor lines is considered best practice, as it increases statistical power, accounts for inter-individual genetic effects, and improves generalizability of findings (Volpato & Webber, 2020; Brunner et al., 2022). However, we note that adding donors is not always feasible in studies involving complex, long-term differentiations, particularly considering the limitations of BSL-4 work.

Our original design included additional iPSC donor lines (including a female donor), but technical limitations prevented successful differentiation into mature 3D gut organoids for some lines—an outcome that reflects known and common differentiation variability across iPSC lines (Volpato & Webber, 2020).

Importantly, we used the iPSC-derived organoids as experimental models in our study to establish a novel infection platform. At this stage of model development, genetic donor variations were not the main focus. This is also reflected in other studies on iPSC-derived tissue development, where novel cell and tissue models were established using only one or two donor lines (e.g., Burgess et al., 2024; Mithal et al., 2020).

To mitigate donor limitations and strengthen the robustness of our findings, we employed several strategies:

- Multiple biological replicates (≥3 differentiations) per line to demonstrate within-line reproducibility
- Orthogonal methods (immunofluorescence, IHC, ISH, RNA-seq) to validate infection and spread
- Matched primary human organoids from independent donors to support translational relevance
- A focus on robust, highly penetrant phenotypes (e.g., spatial spread of viral antigen, viral transcript upregulation, cytopathic effects), which are less likely to be donor-specific artifacts

Additionally, increasing technical replicates and utilizing orthogonal approaches can partially compensate for donor limitations, particularly in studies where increasing donor numbers is impractical (Brunner et al., 2022). Their findings also highlight that adding more observations from the same donor improves reproducibility**,** while adding donors improves generalizability—both of which are addressed in our study design to the extent feasible.

Finally, we respectfully note that this study represents, to our knowledge, the first demonstration of EBOV and MARV infection in a human gut organoid platform. Given the absence of human in vivo models (due to the high lethality of EBOV and MARV and ethical constraints), and the known limitations of animal models in recapitulating human intestinal pathogenesis, we believe our findings provide a meaningful and novel contribution to the field, even within the acknowledged donor limitations. Importantly, this work is not intended as a definitive endpoint, but rather as a foundation for future investigations. We hope it paves the way for further refinement, expansion across more donor backgrounds, and application of this platform to explore disease mechanisms, host-pathogen interactions, and therapeutic strategies in a human-relevant context.

2. Even though an increase in antigen positive cells between 1 dpi and 3 dpi in Fig 3 and 6 is visible and an increase in viral transcripts in Fig 5, a successful dissemination of infectious virus cannot be fully concluded from the data. Quantification of disseminated virus (infectious titers) in the supernatant of the organoids is missing and absolutely needed to validate the statement of successful viral dissemination.

**Response:** We agree with the Reviewer that direct quantification of infectious virus in the supernatant would provide additional support for productive viral replication and dissemination. However, due to BSL-4 biosafety constraints and the design of the original experiments, supernatants were not collected or stored, and thus retrospective analysis is not feasible.

In addition, standard viral titration assays are technically challenging to apply in 3D organoid systems. The semi-enclosed architecture of organoid lumens, along with extracellular matrix components, often limits the release and recovery of virus into the surrounding media. As such, tissue-based approaches (e.g., RNA in situ hybridization and immunohistochemistry) are better suited to assessing viral spread in this model.

Despite the absence of infectious titers, we respectfully argue that the current dataset provides strong, multi-dimensional evidence for intra-organoid viral dissemination:

- Immunohistochemistry (Fig. 3 and 6) reveals a time-dependent increase in viral antigen-positive cells, indicating spread beyond the initially infected population.
- RNA in situ hybridization (ISH) confirms the spatial distribution of viral RNA throughout the organoid, consistent with infection extending to distal regions.
- Bulk RNA-seq (Fig. 5) shows a marked increase in viral transcript abundance over time, supporting active replication and spread.

These findings are concordant across multiple biological replicates and independent methodologies, reinforcing the robustness of our conclusions.

In response to the reviewer’s concern, we have revised the manuscript to more precisely state that our findings provide evidence of *intra-organoid viral spread* rather than definitive confirmation of infectious virus release into the supernatant.

To address the reviewer’s comment, we have revised the Results section to more accurately reflect our findings. The revised sentence now reads:

“Together, the increase in antigen-positive cells, broader spatial distribution of viral RNA, and rising viral transcript levels support intra-organoid viral spread, although direct measurement of infectious virus release into the supernatant was not performed.”

We have also added the following statement to the Limitations section to acknowledge this limitation and outline future directions:

“*A limitation of the current study is the absence of infectious viral titer measurements from organoid supernatants. Due to biosafety constraints and the nature of 3D organoid cultures, viral release into surrounding media is often restricted. Future studies incorporating optimized sampling strategies and viral titration assays will be essential to directly quantify extracellular infectious virus and further validate this model of gastrointestinal infection.”*

3. It is unclear and not mentioned in the M&M section how the MOI was calculated to infect the organoids, since all organoids have a different number of cells. Furthermore, an MOI of 10 seems to be rather high compared to standard 2D cell culture experiments. The authors need to justify the physiological relevance of using such a high MOI and discuss why a lower MOI was not an option?

**Response:** We thank the reviewer for raising this important point. To clarify, the MOI was calculated by dissociating organoids from three representative wells of a 12-well plate into a single-cell suspension, then averaging the cell counts to account for variability in organoid size and number between wells. The total infectious units were calculated using the formula:

Average cells/well x MOI x number of wells to be infected

Given the viral titer (TCID_50_), we then determined the volume of virus needed to achieve the desired MOI.

We would like to emphasize that the used MOI depends on the scientific question. For example, we routinely use a high MOI of 10 for bulk RNA-seq experiments to achieve high infection rates and reduce cell-to-cell variation. Regarding the intestinal organoids, an MOI of 10 was necessary to achieve robust and consistent infection in 3D organoids, which present diffusion barriers due to their structure. Importantly, we tested a range of MOIs (0.1, 10, 50, 100) in primary cell derived colonic organoids, as shown in Figure S4. These data demonstrate that lower MOIs resulted in minimal infection, while higher MOIs produce more robust and consistent infection levels. Based on these findings, we selected MOI 10 as a balance between efficiency and physiological relevance.

We have now clarified this approach in the Materials and Methods.

4. In Figure 3 D the infection rate of EBOV infected HCOs was quantified using flow cytometry, but a similar quantification is lacking for MARV-infected HCOs and for the HIOs. Why? The authors should add a quantification to assess infection rate, especially since quantification of virus in the supernatant or in “lysed” organoids is lacking.

**Response:** While flow cytometry was initially used to quantify infection in EBOV-infected HCOs as (Figure 3D), we were not able to extend this approach to MARV-infected HCOs, probably because the used antibodies did not work for FACS. Notably, all samples produced in the BSL-4 lab go through harsh inactivation procedures which might affect downstream applications. We repeatedly tried to establish FACS analysis for MARV samples without success. This limitation precluded accurate quantification of MARV infection by FACS.

To ensure consistent quantification across the infections, we subsequently implemented IHC-based quantification, which provided a reproducible and spatially resolved measure of infection within the 3D organoid architecture as shown in Figure 3I and 3K.

5. Figure 3 I, K: How was the immune-reactive tissue quantified?

**Response:** The respective information was added to the Methods section.

6. For the determination of differentially expressed genes upon infection “mock- infected control” were used. However, it is unclear what these are exactly. Since residues, like signaling molecules, cytokines, chemokines etc., from the virus propagation and purification can affect sensitive experiments like these unrelated to the actual viral infection, conditioned media (e.g UV-light/ irradiation virus inactivated media) should ideally be used. The authors should elucidate and ideally discuss limitations of their mock control if conditioned media was not used.

**Response:** In our study, “mock-infected” organoids were treated with media alone, processed identically to infected samples—including dissociation, incubation, Matrigel re-embedding, inactivation, and downstream handling—but without the addition of virus. This has now been clarified in the Materials and Methods.

While we understand the rationale for using inactivated virus or conditioned media as controls, these approaches introduce their own limitations. Specifically, UV- or chemically inactivated viruses can still bind cellular receptors and elicit host responses, including stress and immune activation, in the absence of productive infection. This could confound interpretation of transcriptional data. In fact, we previously showed that gamma-irradiated EBOV induces a TLR-triggered innate immune response in macrophages.

Given these considerations, and in line with common practice in the field, we chose media-only mock controls to avoid introducing non-specific stimuli that may obscure virus-specific host responses.

7. What was the minimal RPKM cut off value for the analysis of DEG?

**Response:** In our analysis, we filtered genes based on raw read counts. Specifically, genes with fewer than 10 read counts across all samples were excluded from downstream differential expression analysis. This count-based filtering approach is commonly used to reduce noise from lowly expressed genes and improve statistical power in RNA-seq analyses. We have now clarified this filtering criterion in the Materials and Methods section by adding the following:

“*Genes were filtered based on the standard edgeR filtration method using the default parameters for the “filterByExpr” function, which excludes genes with low expression across all samples. Specifically, genes with fewer than 10 counts were removed from the dataset prior to differential expression analysis.”*

8. The statement that EBOV and MARV infection leads to extensive disruption of epithelial structure is overinterpreting the RNA seq data. In order to support this conclusion, this needs to be verified on a protein/phenotype basis. How does the epithelial layer look like in IFA of infected organoids?

**Response:** We agree that transcriptomic data alone does not fully confirm structural disruption. To address this, we have revised the text to more cautiously interpret the RNA-seq findings in the context of epithelial remodeling.

In support of structural perturbation, we direct the reviewer to Figures 3 and 6, where immunofluorescence analysis shows widespread viral dissemination by 3 dpi in both HCOs and HIOs, alongside maintained, but visible strained, epithelial architecture marked by CDX2. Figure S1 also shows similar results marked by intestinal marker Villin 1. Additionally, Figure 2 highlights pronounced cytopathic effect by 7 dpi for HCOs, including organoid collapse and visible morphological changes.

While our IHC analyses focused on viral antigen detection, they further show dense viral staining and altered organoid morphology by 3 dpi. Finally, the forskolin swelling assay in Figure 7 revealed abnormal morphology of infected organoids, including darkening of the luminal space and roughened epithelial edges. These phenotypic changes support the inference that filoviral infection perturbs epithelial structure, complementing the transcriptional signatures of hypoxia and EMT activation. We have now clarified this point in the revised text.

To address this, we have revised the relevant sentence in the Introduction to more accurately reflect the findings and incorporate morphological evidence. This sentence now reads as follows:

“*Additionally, infections with both viruses led to the significant dysregulation of genes involved in apical surface structure and tight junction integrity, correlating with increased viral replication and morphological changes indicative of epithelial disruption.”*

Similarly, in the Discussion, the original statement has been revised to:

“*Together, these findings suggest that filovirus infections perturb epithelial structure and function, as supported by both transcriptional signatures and immunofluorescence evidence of disrupted epithelial organization (Figures 2 and 3).”*

These revisions provide a more cautious and evidence-integrated interpretation consistent with the reviewer’s comments.

9. Why does the expression of gut markers increase in the 3 dpi mock HCOs? Shouldn’t they stay consistent in the mock infection?

**Response:** This is an interesting and important point raised by the Reviewer. The expression of gut-specific genes is known to be highly dynamic in intestinal organoid cultures, including under mock-treated conditions. The increased expression of gut epithelial markers observed at 3 dpi in mock-infected HCOs is consistent with the expected progression of organoid maturation and differentiation over time. This reflects normal biological development rather than an experimental artifact. To ensure accurate interpretation of infection-specific effects, mock-infected controls were included at each time point (e.g., 1 dpi and 3 dpi), allowing for time-matched comparisons that account for baseline developmental changes in the system.

Specifically:

1. **Ongoing differentiation in iPSC-derived intestinal organoids:** These organoids are dynamic, self-organizing systems that model aspects of intestinal development. Even in the absence of infection or external stimulation, they undergo progressive differentiation over time, transitioning from a proliferative, stem-cell–like state toward more mature epithelial cell types. This temporal maturation is marked by increased expression of lineage-specific markers for enterocytes, goblet cells, and enteroendocrine cells (Kilik et al., 2021).
2. **Response to culture conditions and medium composition:** The mock-treated organoids are cultured under conditions that continue to promote epithelial organization and maturation, including exposure to defined growth factors and

extracellular matrix components. This environment supports differentiation trajectories that lead to elevated expression of gut epithelial markers over time. For example, (Klein et al., 2025) demonstrated that even in mock-treated iPSC-derived small intestinal organoids, markers such as FABP1, MUC2, ALPI, and TFF3 increase in expression during early culture time points, reflecting the expected transition from proliferative to differentiated states.

3. **Establishment of complex epithelial architecture and biological variability:** Organoids develop from simple spheroids into complex 3D structures with crypt-like budding, lumen formation, and regionalized cell types. These processes involve dynamic transcriptional changes in structural and functional epithelial markers. Additionally, sex- and strain-specific differences in gene expression during organoid development have been reported in other systems, such as murine small intestinal organoids (Jang et al., 2025), further illustrating how gut marker levels can shift even under control/mock conditions.

Importantly, we emphasize that all infection-driven transcriptional changes are assessed relative to these mock-infected samples at the same time point, thereby controlling for the inherent maturation trajectory of the organoids.

To make this point clearer for the readers, we added the following section to the discussion section:

“*It is important to note that iPSC-derived intestinal organoids undergo ongoing differentiation and maturation during culture, even in the absence of infection. This temporal progression—from a proliferative, stem-like state toward mature epithelial cell types—is marked by increased expression of gut lineage-specific markers such as FABP1, MUC2, ALPI, and TFF3 (Kilik et al., 2021; Klein et al., 2025). Additionally, organoid development is influenced by culture conditions, including growth factors and extracellular matrix components, which support continued epithelial maturation. These changes are consistent with previously reported increases in enterocyte and goblet cell markers during early organoid development (Klein et al., 2025) and sex- or strain-specific differences in gene expression during differentiation (Jang et al., 2025). To account for these dynamics, all infection-induced gene expression changes in our study were analyzed relative to time-matched mock-infected controls, ensuring that the effects attributed to filoviral infection are distinct from baseline developmental processes.”*

10. The authors find a greater epithelial expression of innate immune markers upon MARV infection compared to EBOV infection. Why is that?

**Response:** We thank the reviewer for this important question. Our finding that MARV infection induces stronger epithelial innate immune responses than EBOV aligns with previous reports in other cell types. This difference is probably attributed to distinct viral immune evasion strategies: EBOV VP35 seems to more effectively antagonize RIG-I-like receptor signaling and type I IFN production than MARV VP35, due to structural and RNA-binding differences in their IFN inhibitory domains (Edwards et al., 2016). Additionally, MARV and EBOV use different strategies to antagonize IFN signaling, which might also contribute to the observed differences. Together, the less potent immune evasion mechanisms of MARV likely contribute to the heightened innate immune activation observed in our epithelial model. We have added a sentence to the Results section to clarify this point, which now reads:

“*This difference is likely due to distinct immune evasion strategies: MARV VP35 is less effective at antagonizing RIG-I-like receptor signaling and type I interferon production than EBOV VP35, owing to structural and RNA-binding differences in their interferon inhibitory domains.”*

11. The authors use FISH to quantify mRNA expression of innate immune markers in the organoids wanting to validate findings from bulk RNA seq. However, a quantification based on protein expression instead of mRNA, especially in the context of imaging tissues, is a much better readout and validation and needs to be added to validate the claims. Additionally, the description of Fig. S3 is inconclusive showing one point, saying two independent infections (n=3). How many images were analyzed to rule out using one “good-looking” image?

**Response:** We appreciate the reviewer’s suggestion regarding protein-level validation. However, we chose FISH because it allows highly specific spatial localization of mRNA within the 3D tissue context of our organoids, which is often challenging with antibody staining due to issues such as background signal and cross-reactivity. Additionally, we have well-validated probes for the key innate immune markers, ensuring sensitive and specific detection. While protein expression is valuable, mRNA detection by FISH provides a robust and reliable readout that aligns closely with our bulk RNA-seq data. Due to the current limitations in validated antibodies for these markers in our organoid system, we believe our approach is appropriate for this study.

Regarding Figure S3, we apologize for any lack of clarity in the description. The quantification presented is based on images from two independent infections, with three biological replicates each (n=3). For each replicate, we analyzed multiple images (at least 5 fields of view per sample), and quantification represents the average across these fields. The figure legend has been revised and methods section to explicitly state the number of images analyzed to clarify that the results are not based on single “good-looking” images but on robust, reproducible sampling.

12. the entire manuscript is very wordy and needs editing for a reader to stay engaged. It is very hard to read.

**Response:** We have revised and trimmed the Discussion section to improve clarity and readability.

**Part III – Minor Issues: Editorial and Data Presentation Modifications**
Please use this section for editorial suggestions as well as relatively minor modifications of existing data that would enhance clarity.

We appreciate the reviewers’ careful reading of the manuscript and have addressed all minor editorial and data presentation issues.

Reviewer #1: Figure 3J: the MARV 3 dpi panel reads EBOV
Figure 8D: Any potential statistics?

**Response:** Statistical analyses, including p-values, have now been added to Figure 8D to support the conclusions drawn from the data.

Reviewer #2: 1. In Fig. 5, I was surprised that significant gene expression changes were observed in mock organoids from day 1 to 3, particularly in the gut markers which are highly upregulated. This suggests that the organoids were incompletely differentiated when they were initially infected. While I don’t expect you to redo the study with fully differentiated iPSC- derived organoids. You should at least discuss this result.

**Response:** We thank the reviewer for this important observation. The transcriptional changes observed in mock-infected organoids between day 1 and day 3 suggest that infection occurred during an ongoing phase of epithelial maturation, in line with what we discussed in our response to Part II Q9. While our intestinal organoid platform is composed solely of epithelial cells and lacks immune components, it is well established that epithelial maturation involves changes in barrier integrity, cell polarity, and intrinsic antiviral signaling. A more fully differentiated epithelium could influence viral entry dynamics, replication kinetics, and epithelial resilience to cytopathic effects. Moreover, distinct epithelial cell types (e.g., enterocytes vs. goblet cells) may vary in their permissiveness to EBOV and MARV infection. These considerations highlight the importance of developmental timing in host-pathogen interactions and suggest that future studies exploring infection across maturation stages may provide additional insight. See alos our response to

2. In Fig. 7, is there a reason why only one time point is used? It is not clear whether this normalized data, wherein one data point is normalized to mock reflects a decrease in reads in mock cells or an increase in reads in infected cells (see point 1). This caveat should be discussed.

**Response:** We thank the reviewer for raising this important point. The gene expression data presented in Figure 7 are normalized to match mock-infected controls for each condition, ensuring clarity regarding the directionality of observed transcriptional changes between infected and control samples. The focus on 3 dpi for HIOs was informed by prior time-course experiments in HCOs, which revealed maximal virus-specific transcriptional divergence at this time point. This rationale is detailed in the Results section, where we describe how the HIO RNA-seq analysis builds upon earlier findings in HCOs to capture later-stage, virus-specific responses.

3. Fig. 8C needs statistical significance.

**Response:** We appreciate the reviewer’s comment. Figure 8C presents representative images of HIOs before and after forskolin stimulation to illustrate the OrganoSeg™-based segmentation and quantification approach. These images are not intended to show quantifiable results or statistical comparisons; rather, they serve to visually demonstrate the imaging workflow described in the methods. Statistical analysis of the forskolin response is provided in the corresponding quantification panel (Figure 8D) and described in the text.

Reviewer #3: 1. l. 52: Is the use of both comma and em dash here correct? Please double check.

**Response:** The punctuation has been corrected in the revised manuscript.

2. l. 58: “provide insights into the transmission” and pathogenesis?

**Response:** We agree that pathogenesis is an important aspect of filovirus disease and have revised the sentence to include it for completeness.

3. Figure 1: Brightfield images in D are very difficult to see due to the small size. Can the size be increased, or a smaller window used so individual cells are easier to see?

**Response:** We appreciate the reviewer’s suggestion. Panel 1D is intended to provide a representative view of gross morphology at the progenitor stage throughout the directed differentiation, consistent with standard practice in the field. As these are not analytical images, we have retained the current layout to preserve figure integrity and consistency across the manuscript. We have, however, confirmed that the images are of sufficient resolution at journal scale for visualizing overall structure, and we’ve clarified the illustrative nature of this panel in the figure legend.

4. l. 121: please first introduce dpi upon first usage in the manuscript.

**Response:** We have now defined dpi at its first occurrence in the manuscript to improve clarity for readers.

5. Figure 2: 7 dpi images for HIOs are missing?

**Response:** Images were acquired at 1, 3, and 7 dpi for HCOs. Based on the extent of the cytopathic effect observed in HCOs at 7 dpi, we did not image HIOs at this point, as we anticipated similar or greater structural collapse that would preclude acquisition of representative images. In addition, organoids at 7 dpi were not used for downstream analyses due to the severity of virus-induced damage. We have now added the following sentence to the Results section for clarification:

“*Due to the extent of virus-induced damage, organoids at 7 dpi were not included in downstream analyses.”*

6. l. 207: “1- and 3-dpi” should be without hyphenation since it has been written without it in the rest of the manuscript.

**Response:** We have corrected the hyphenation at line 207 to maintain consistency with the rest of the manuscript.

7. Figure 4 G-H: to increase readability consider erasing the “Hallmark” in front of every hallmark name. These graphs are very small. Consider only displaying the significant ones in the main figure to further increase readability of these types of figures in general.

**Response:** To address the reviewer’s concern regarding figure readability, we have removed the repeated “Hallmark” label from each pathway name in Figures 4G-H and Figure 7C to declutter the graphs and enhance clarity. We want to highlight that “Hallmark” is not a generic term but specifically refers to the curated Hallmark gene sets from the Molecular Signatures Database (MSigDB), a widely recognized and standard resource in gene set enrichment analysis (GSEA). This terminology is important for accurately describing the analysis performed.

8. Figure 5: Not readable and hard to draw conclusion from in its current state. Consider highlighting certain genes and only showing those and breaking them up into groups while showing an even bigger image of the full set in the supplement.

**Response:** We thank the reviewer for this feedback. After careful consideration among co-authors, we chose to retain the current figure layout, which includes the full Hallmark heatmaps on the left and corresponding curated gene sets on the right (e.g., ISGs, diarrheal pathogenesis-related genes and hypoxia-related genes). To aid readability, we had annotated selected genes of interest in larger font with arrows, which correspond to genes highlighted throughout the Results and Discussion sections. This format was designed to balance pathway-level overview and gene-level resolution. Additionally, to further enhance accessibility, we have deposited the full RNA-seq datasheets in the Gene Expression Omnibus (GEO) under accession numbers GSE298600 and GSE300073, with access tokens utexykmulfoldkl and uxozcygstbmdlaf, respectively. These data allow interested readers to explore the full transcriptomic profile in greater detail.

9. ll. 862-863; ll. 898-899; ll. 957-958: safe spaces between number and unit to not separate them in between two lines.

**Response:** We have adjusted the formatting so that the number and unit now remain on the same line in each of the mentioned instances.

10. l. 878; l. 887: “1X PBS” should be “1× PBS”.

**Response:** Since we exclusively used “1x” PBS throughout the study, we have removed “1x” for consistency.

11. l. 929 : Is the version number missing for MiniNet or is the line intentional?

**Response:** We would like to apologize for the confusion. Since the version number was already provided in the section above (HALO software (v4.05107.357; Indica Labs, Inc., Corrales, NM)) and we wanted to avoid redundancy, we did not reinstate the version number. However, for clarity, we have now removed the confusing empty line.

12. l. 942f. “1- and 3- days dpi” should be “1 and 3 dpi”.

**Response:** This has been corrected as suggested.

13. l. 951: “10-minute” should be “10 minute”.

**Response:** “10-minute” is correct. This is a compound adjective.

14. l. 989: Throughout the M&M section spaces between number, times and g was written (200 × g), but here no space. Please decide on one across the entire section.

**Response:** We have reviewed the Methods section and ensured consistent formatting for all units (e.g., “× g”) throughout.

15. Table 1: The title of table one is inaccurate as the table not only lists antibodies but antibodies, dyes, and reagents. Please correct.

**Response:** The table title has been updated to accurately reflect the listed materials.

16. The table design of table 3 is a bit unfortunate since, when printed does not show the lines making it hard to decipher and read. A lot of the words and names are split in weird ways making it harder to read. Think about switching this table into landscape to make it readable.

**Response:** We have reformatted Table 3 in landscape orientation and adjusted the margins to improve readability and prevent word splitting in printed form.
