# Supplementary material for: Filovirus infection disrupts epithelial barrier function and ion transport in human iPSC-derived gut organoids
Source: PLoS Pathog. 2025 Nov 24;21(11):e1013698. doi: 10.1371/journal.ppat.1013698 (PMC12698023; doi:10.1371/journal.ppat.1013698)
Supplement: Attachment — Submitted filename: response letter 2.docx [file ppat.1013698.s012.docx]

**Rebuttal letter for second resubmission.**

**Figure resubmission:**

While revising your submission, we strongly recommend that you use PLOS’s NAAS tool (<https://ngplosjournals.pagemajik.ai/artanalysis>) to test your figure files. NAAS can convert your figure files to the TIFF file type and meet basic requirements (such as print size, resolution), or provide you with a report on issues that do not meet our requirements and that NAAS cannot fix.

After uploading your figures to PLOS’s NAAS tool - <https://ngplosjournals.pagemajik.ai/artanalysis>, NAAS will process the files provided and display the results in the "Uploaded Files" section of the page as the processing is complete. If the uploaded figures meet our requirements (or NAAS is able to fix the files to meet our requirements), the figure will be marked as "fixed" above. If NAAS is unable to fix the files, a red "failed" label will appear above. When NAAS has confirmed that the figure files meet our requirements, please download the file via the download option, and include these NAAS processed figure files when submitting your revised manuscript.

**Response:** We used the NAAS tool to meet the figure requirements of Plos Pathogens. We have replaced Figures 1–8 with NAAS-approved versions and hope that they now satisfy the quality requirements of Plos Pathogens.

**Reproducibility:**
To enhance the reproducibility of your results, we recommend that authors of applicable studies deposit laboratory protocols in protocols.io, where a protocol can be assigned its own identifier (DOI) such that it can be cited independently in the future. Additionally, PLOS ONE offers an option to publish peer-reviewed clinical study protocols. Read more information on sharing protocols at <https://plos.org/protocols?utm_medium=editorial-email&utm_source=authorletters&utm_campaign=protocols>

**Response:** To enhance reproducibility, we have deposited comprehensive protocols for the differentiation of HIOs, HCOs, and PCOs (part 1; DOI [10.17504/protocols.io.e6nvw44p2lmk/v1](https://dx.doi.org/10.17504/protocols.io.e6nvw44p2lmk/v1)) and standardized infection of human gut organoids with filoviruses (part 2; [10.17504/protocols.io.ewov111jpvr2/v1](https://dx.doi.org/10.17504/protocols.io.ewov111jpvr2/v1)). These protocols are now referenced in the revised manuscript.
